# Association between Health-Related Physical Fitness and Respiratory Diseases in Adolescents: An Age- and Gender-Matched Study

**DOI:** 10.3390/ijerph18126655

**Published:** 2021-06-21

**Authors:** Jeong-Hui Park, Myong-Won Seo, Hyun Chul Jung, Jong-Kook Song, Jung-Min Lee

**Affiliations:** 1 Department of Physical Education, Kyung Hee University (Global Campus), 1732 Deokyoungdaero, Giheung-gu, Yongin-si 17014, Gyeonggi-do, Korea; jeonghee@khu.ac.kr; 2 Department of Taekwondo, Kyung Hee University (Global CAMPUS), 1732 Deokyoungdaero, Giheung-gu, Yongin-si 17014, Gyeonggi-do, Korea; myongwonseo@khu.ac.kr (M.-W.S.); jksong@khu.ac.kr (J.-K.S.); 3 Department of Coaching, Kyung Hee University (Global Campus), 1732 Deokyoungdaero, Giheung-gu, Yongin-si 17014, Gyeonggi-do, Korea; jhc@khu.ac.kr; 4 Sports Science Research Center, Kyung Hee University (Global Campus), 1732 Deokyoungdaero, Giheung-gu, Yongin-si 17014, Gyeonggi-do, Korea

**Keywords:** physical fitness, respiratory diseases, physical activity, sedentary behavior, adolescents

## Abstract

The current study examined the differences in health-related physical fitness (HRPF), physical activity (PA), and sedentary behavior (SB) between adolescents with and without ongoing respiratory diseases (RD). This study’s participants were from 12 to 15 years old (7th–10th grade) in South Korea. Adolescents with RD were selected through RD-related questions (i.e., asthma, rhinitis, sinusitis, and bronchitis) (*n* = 139); in contrast, adolescents without RD, randomly selected from the general group, responded to any health problem-related questions as “No” (*n* = 139). HRPF was measured based on the FITNESSGRAM and EURO FIT test batteries and the measurements of HRPF included cardiovascular endurance, muscular strength, muscular endurance, flexibility, and body composition. All statistical analyses were conducted by SPSS 25.0, and the independent *t*-test was used to compare the HRPF and PA between the two groups. Moreover, the measured HRPF was compared with a series of analyses of three-way ANOVAs (age × gender × group). Adolescents with RD had a positive association with less participation in PA (*p* < 0.05; RD: 3081.81 ± 4793.37; general: 2073.64 ± 3123.47) and with more time spent on SB (above 12 h per week: RD group (38.85%) and general group (33.09%)). Furthermore, adolescents in the RD group showed significant effects on all components of HRPF (*p* < 0.05). Our study confirmed that HRPF is an essential predictor of adolescents’ health outcomes, especially for those with RD. We suggest that increased HRPF can be an effective treatment for respiratory diseases in adolescents, and health practitioners should pay more attention to helping adolescents with RD to gain or maintain high HRPF.

## 1. Introduction

Adolescence is a preliminary stage in preparing for an adult’s role, which involves undergoing various changes, such as physical, psychological, and social development processes. Therefore, adolescence is when significant health-related behaviors are determined [1]. In particular, health-related physical fitness (HRPF) has been identified as a crucial predictor of health outcomes in adolescence, as well as in adulthood [2,3]. The components of physical fitness, including cardiovascular endurance, muscular strength and endurance, flexibility, and body composition, are associated with risk factors of cardiovascular diseases and musculoskeletal health [4,5,6]. Improvement in HRPF during school age and adolescence is important for maintaining a healthy lifestyle in adulthood. For instance, adolescents with low HRPF more likely to be overweight and obese rather than those with high levels of HRPF [7] and this unfavorable condition links to obesity in adulthood, which results in adverse health outcomes in later life [8,9].

Additionally, some studies have reported that there is a strong association between HRPF and physical activity (PA) [10,11]. One study examined the correlation between HRPF and PA in adolescents and the results indicated that there are obvious interconnections between HRPF and moderate-to-vigorous physical activity (MVPA), and adequate HRPF for adolescents could be derived from sufficient MVPA [12]. Pedro and colleagues’ study demonstrated that adolescents’ HRPF is not only associated with MVPA, but also sedentary behavior (SB), so promoting MVPA and reducing prolonged SB should be considered first to improve HRPF [13]. Therefore, adolescents’ HRPF, PA, and SB are closely related by their underlying causal factors and should be considered together when their health is measured.

Despite accumulated scientific evidence on the importance of HRPF and PA in adolescents, their HRPF and PA have been declining annually over the past decade [14], and similarly, this finding is consistent with Asian adolescents’ HRPF and PA studies [15,16,17]. Poor HRPF and low participation in PA has eventually led to an increase in the prevalence of obesity and respiratory diseases (RD) (i.e., asthma, rhinitis, and sinusitis) because of exacerbation of the pulmonary function [18,19]. Specifically, typical RD includes asthma and rhinitis, and asthma is known as a chronic lower RD with various symptoms such as hypersensitivity of the airway, phlegmatic cough, and chest compressions [20]. Moreover, rhinitis is an upper RD with symptoms such as itching, runny nose, and sneezing [21], and the symptoms are closely correlated with other RDs, such as sinusitis and bronchitis [22,23].

According to the International Study of Asthma and Allergies in Childhood (ISAAC), the number of adolescents with RD has increased and this phenomenon is expected to gradually increase with an additional 400 million adolescents with RD by 2025 [24,25]. Although there is little doubt that the cause of the increased prevalence of RD is multifactorial (i.e., environment, air pollution, and genetics) [26,27], there is a growing body of literature that implicates lifestyle changes, specifically decreased PA and HRPF, as being a strong contributor to the increase in RD [28,29,30]. As an extension of the aforementioned studies, a recent study conducted by Park and colleagues reported that adolescents with RD participate less in PA and maintain SB longer than those who without RD, and they also have a higher BMI than general adolescents [31].

The overwhelming majority of studies have demonstrated that increasing HRPF, engaging in regular PA, and reducing SB has the major health benefit of alleviating RD, but there has not yet been an empirical investigation into different HRPF, PA, and SB levels of adolescents with ongoing RD compared to general adolescents. Therefore, the primary aim of the study was to investigate the differences in HRPF, PA, and SB between adolescents with and without ongoing RD. Additionally, the secondary aim was to examine the impacts of RD on HRPF, PA, and SB among different ages and genders in comparison to general adolescents.

## 2. Methods

### 2.1. Design

The participants were recruited from each city in South Korea using stratified sampling procedures based on the geographic regions, school districts, school type, and gender distribution of each city. Specifically, one or more school districts were selected within each city. Data from 1686 adolescents were collected and all participants aged 12–15 years (7th–10th grade) with a health status that allowed participation in physical fitness tests were eligible.

### 2.2. Study Participants

One thousand six hundred and eighty-six Korean adolescents participated in the HRPF test and responded to the questionnaire in the current study. Among these, adolescents with RD were selected through health-related questions (health assessment survey questions), and all adolescents with RD were matched for age and gender with adolescents without RD. Adolescents in the RD group (*n* = 139) either answered the asthma-related questions (i.e., “Are you currently suffering from asthma?”/“Have you ever been to a hospital because of asthma?”) with “Yes” (*n* = 85) or reported that they had other RDs besides asthma (i.e., rhinitis, sinusitis, and bronchitis) (*n* = 54). In contrast, adolescents, randomly selected to the general group, responded to any health problem-related questions such as above with “No” (*n* = 139). Before the study, we provided a complete written explanation regarding the study’s purpose, procedure, and possible occurrence of discomfort and risks, and we received written informed consent from each participant’s parent or legal guardian. All participants were aware that they could withdraw at any time without any prejudice. This study was approved by the Institutional Review Board (IRB) of Mahidol University (MU-IRB2014/029.1302).

### 2.3. Measures

#### 2.3.1. Health-Related Physical Fitness (HRPF)

HRPF was measured based on the FITNESSGRAM [32] and EURO FIT [33] test batteries, both of which have demonstrated good reliability and validity for use in adults, as well as adolescents [34,35]. The measurements of HRPF included cardiovascular endurance, muscular strength, muscular endurance, flexibility, and body composition, and the entire testing process was conducted under the supervision of well-trained assistants. (1) *Cardiovascular endurance* was evaluated using a 15 m progressive aerobic cardiovascular endurance run (15 m PACER) and was performed once. The 15 m PACER is an alternative way to measure cardiovascular endurance and an easy-to-measure for a limited gymnasium, as evidence suggests that it provides a similar profile of the adolescent’s cardiovascular endurance compared to the 20 m PACER [36]. (2) *Muscular endurance* was evaluated through a 1 min sit-up test and performed once. (3) *Muscular strength* was evaluated using a hand-grip strength dynamometer with accuracy to the nearest 0.1 kg (Takei Scientific Instruments Co. Ltd., T.K.K. 5101 Grip D, Tokyo, Japan). The measurements, left-hand and right-hand grip strength, were taken two times and the average score was calculated. (4) Flexibility was measured using an Acuflex I modified flexibility sit-and-reach test box (PSYMTEC, Madrid, Spain). As muscular strength measurements, the sit-and-reach test was also measured two times for the left and the right leg, and the average scores were calculated from the measured test scores. (5) *Body composition* was measured by biometric impedance analysis (BIA, Tanita, TBF-543, Japan), and height (SECA S-208M, the United States) and weight (TANITA, BC-581, Japan) were measured directly. Additionally, body mass index (BMI) was calculated by dividing the weight (kg) by the square of the height (m^2^).

#### 2.3.2. Self-Reported Physical Activity (PA)

Self-reported PA was measured using the International Physical Activity Questionnaire-Short Form (IPAQ-SF) and the Korean version of the IPAQ-SF has demonstrated high reliability and validity [37]. All participants reported frequency (i.e., days), duration (i.e., minutes), and intensity (i.e., moderate and vigorous) of their participation in PA over the past seven days. PA was calculated by “metabolic equivalent task (MET) level × minutes × number of days per week” for each intensity, with 4.0 METs for moderate physical activities and 8.0 METs for vigorous physical activities. Meanwhile, moderate-to-vigorous-intensity PA was calculated by the sum of METs calculated from moderate- and vigorous-intensity PA per week.

#### 2.3.3. Self-Reported Sedentary Behavior (SB)

Self-reported SB was estimated by the interviewer-administered Adolescent Sedentary Activity Questionnaire [38], and the participants were asked how many hours they sit for study and leisure time during a week. SB included sitting time in their house, school, and private institute, and SB for leisure included any sedentary time spent, for example, watching TV or playing video and online games, except for studying.

### 2.4. Statistical Analysis

The demographic information (i.e., gender, age, height, and weight) and SB of the participants were summarized by descriptive statistics in SPSS version 25.0 (IBM, Chicago, IL, USA). The independent *t*-test was used to compare the HRPF (i.e., cardiovascular endurance, muscular endurance, muscular strength, flexibility, and body composition) and PA between the two groups. Furthermore, the measured HRPF was compared with a series of analyses of three-way ANOVAs (age × gender × group), and post-hoc analysis was performed using Bonferroni’s correction among age, gender, and groups. Cohens’ *d* (small ≥ 0.2, medium ≥ 0.5, and large ≥ 0.8) was used to assess the standardized difference in PA, SB, and HRPF between the RD and general groups. The least-squares means and standard errors were estimated within the model and the overall effects were examined with the standard *F*-test.

## 3. Results

The demographic characteristics and anthropometric measurements (i.e., height, weight, and BMI) were analyzed by descriptive statistics as frequencies and proportions (Table 1). A total of 278 adolescents participated in the present study and the mean of ages for both groups (i.e., general and RD groups) was 13.38 years; males comprised 60.43% and females 39.57% of the sample. The adolescents’ characteristics (i.e., gender and age) indicated no significant differences between the two groups, because age and gender were matched. No anthropometric measurements (i.e., height, weight, and BMI) showed significant differences between the two groups.

The results of adolescents’ PA, SB, and HRPF between the general and RD groups are presented in Table 2. Moderate-intensity PA (MPA; *p* = 0.047, Cohen’s *d* = 0.23) and MVPA (*p* = 0.039, Cohen’s *d* = 0.24) indicated significant differences, but vigorous-intensity PA (VPA) showed no significant differences between the two groups (*p* = 0.660, Cohen’s *d* = 0.22). In addition, in SB, adolescents who spent from 6 to 12 h a week sitting down accounted for a higher percentage of those in the general group (46.04%) than those with RD (41.01%), while adolescents who spent more than 12 h a week sitting down accounted for a higher percentage of those in the RD group (38.85%) rather than those in the general group (33.09%). According to the results for HRPF presented in Table 2, RD showed significant effects for each of the components of HRPF, including cardiovascular endurance (*p* < 0.001, Cohen’s *d* = 0.67), muscular endurance (*p* = 0.003, Cohen’s *d* = 0.35), left/right strength (left: *p* = 0.003, Cohen’s *d* = 0.35; right: *p* = 0.006, Cohen’s *d* = 0.33), left/right flexibility (left: *p* = 0.003, Cohen’s *d* = 0.35; right: *p* = 0.013, Cohen’s *d* = 0.29), and body composition (*p* = 0.038, Cohen’s *d* = 0.28).

**Figure 1:**
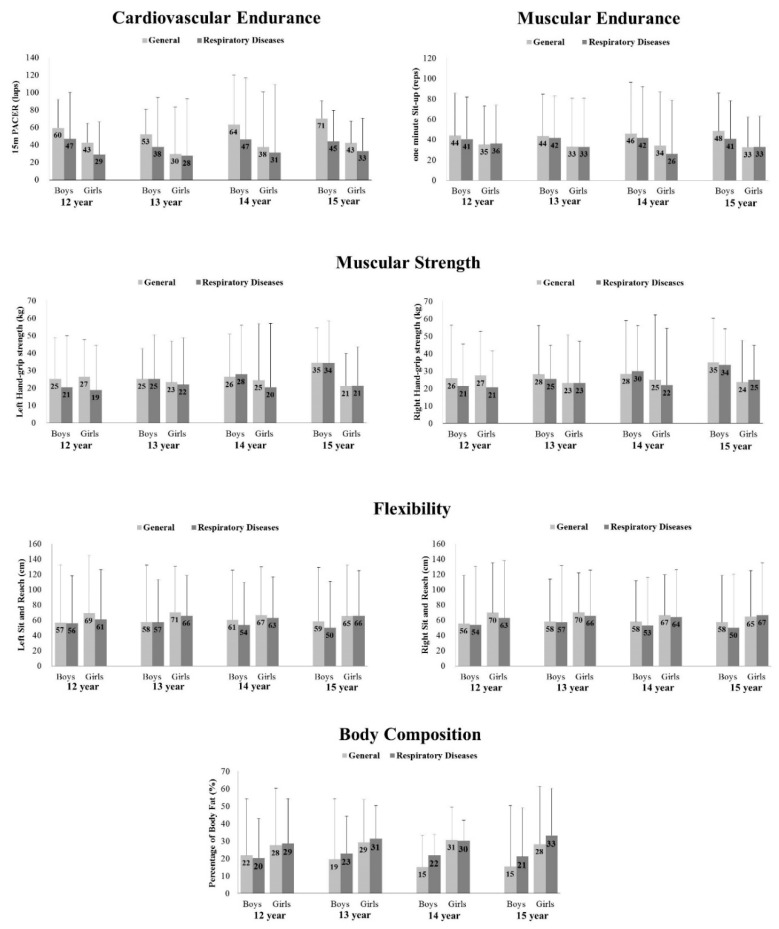
Health-related physical fitness test in aerobic capacity, muscular endurance, muscular strength, muscular flexibility, and body composition. PACER: Progressive aerobic cardiovascular endurance run. The differences in HRPF with their corresponding parameters (i.e., respective standard errors) are presented.

Figure 1 displays the mixed-model 4 × 2 × 2 (age × gender × group) analysis of variance (ANOVA). The interaction of age × gender × group was nonsignificant (*p* > 0.05), but there were significant main effects for group (*p* < 0.05) and gender (*p* < 0.001) in the measured HRPF. Moreover, the effects for age were only revealed in left/right hand-grip (left: *p* < 0.001; right: *p* < 0.001) and PACER (*p* = 0.003). Bonferroni post-hoc tests revealed nonsignificant pairwise differences between gender, age, and group in the components of HRPF (*p* > 0.05), but left/right hand-grip was significantly different in the two-way interaction for age × group (left: *p* = 0.002, *F*(3, 262) = 5.147, partial η^2^ = 0.056; right: *p* = 0.021, *F*(3, 262) = 3.289, partial η^2^ = 0.036). Additionally, there were significant differences in the two-way interaction for age × gender (left: *p* < 0.001, *F*(3, 262) = 14.673, partial η^2^ = 0.144; right: *p* < 0.001, *F*(3, 262) = 8.283, partial η^2^ = 0.087), which yielded a large effect size, and PACER showed a significant difference in the two-way interaction for gender × group (*p* = 0.027, *F*(1, 262) = 4.918).

## 4. Discussion

The purpose of this study was to investigate the differences in HRPF, PA, and SB between adolescents with and without ongoing RD. The prevalence of RD in adolescence is uncertain, both in terms of its mechanism and directionality, and it does not have a life-threatening effect [39]. However, RD problems in adolescence are not only highly likely to continue into adulthood, but also have adverse impacts on quality of life, disability, and productivity [40]. Despite many studies that have emphasized the importance of maintaining appropriate PA and fitness to ameliorate RD [41,42], the current evidence on HRPF in adolescents with RD is ambiguous, and some studies have even demonstrated the outcomes measured by only a specific HRPF of adolescents with RD [43,44].

This study compared HRPF, PA, and SB, as well as anthropometric information, between adolescents with and without RD. The main findings of the current study were that RD (i.e., asthma, rhinitis, sinusitis, and bronchitis) in adolescence is positively associated with HRPF (i.e., cardiovascular endurance, muscular endurance, muscular strength, flexibility, and body composition). Although it is difficult to determine any direct relationships between RD and HRPF, our study confirmed that adolescents with RD not only have significantly lower HRPF than general adolescents, but also less participation in PA and more time spent engaging in SB. Therefore, we speculate that adolescents with RD have a robust association with lower HRPF, less participation in PA, and more time spent engaging in SB.

Specifically, it is worth noting that adolescents with RD had a relatively higher BMI than general adolescents. Although anthropometric information such as body weight and BMI were not significantly different between the two groups, average BMI was higher for both boys and girls in the RD group than those in the general group. Our results are consistent with previous studies [31,45,46], which serve as an opportunity to empower the results of prior studies. Furthermore, the RD group’s adolescents who showed a high BMI participated in significantly lower MVPA (*p* < 0.05) and spent more than 12 h a week sitting down rather than the general group’s adolescents (general: 33.09%; RD: 38.85%). We assume that lower MVPA and more time spent engaging in SB could potentially contribute to the high BMI in adolescents with RD [47]. Most adolescents with RD are known to suffer from uncontrolled and burdensome symptoms caused by RD such as rhinorrhea, sneezing, postnasal drip, daytime fatigue, and sleep disturbance that directly interfere with their daily lives [48,49]. These symptoms reduce quality of life and sustain extreme stress [50], which may have affected the low participation in PA and the increased time spent engaging in SB in adolescents with ongoing RD.

Our study provides novel findings regarding the association between HRPF and RD, cardiovascular endurance, and muscular strength specifically. The PACER test is a well-established indicator of cardiovascular endurance, and this component has been associated with body composition, as well as the risk of all-cause mortality, metabolic diseases, and cardiovascular diseases [51,52]. Meanwhile, muscular strength (i.e., hand-grip strength) is also known as a major risk factor for coronary heart disease, stroke, suicide, and even cancer [53,54,55]. Despite the outcomes of many prior studies, the level of cardiovascular endurance and grip strength of adolescents with RD were significantly lower than those of general adolescents (*p* < 0.001 and *p* < 0.01, respectively), and its outcomes are consistent with Latorre-Román’s findings that demonstrated an association with levels of RD severity and muscular strength [56]. The reason could be because adolescents with RD may still have concerns about exacerbation of their RD during any participation in PA. Since low HRPF has a multifactorial cause (i.e., age, gender, and nutritional status), it cannot be determined whether promoting PA is the only factor that can overcome low HRPF, but increasing participation in regular PA and reducing SB seems to be an effective way to improve their HRPF and prevent other diseases that can be caused by low HRPF [57].

The present study has the following positive strengths and limitations. To the best of our knowledge, no other studies have directly measured the HRPF of adolescents with RD or have compared HRPF between adolescents with and without RD. Furthermore, the main findings of this study add to the existing literature on the association between RD and adolescents’ health-related behaviors (i.e., MVPA, SB, and body weight status) and also provide new insights regarding the factors contributing to RD, such as HRPF and PA. However, since this study did not consider the severity of respiratory diseases, future studies need to scrutinize how the severity of respiratory diseases affects HRPF. Additionally, the participants in this study were limited to Korean adolescents. Therefore, it is necessary to examine more evidence with various ethnicities and adequate sample sizes from the same perspective.

## 5. Conclusions

This study investigated the association between HRPF, PA, and SB, comparing adolescents with RD to those without. The results support that adolescents with RD engage less in PA and spend more time in SB than general adolescents, and is has been evidently confirmed that HRPF is an essential predictor of adolescents’ health outcomes—especially for those with RD. The strategy that can be pursued through this study is brief but exciting, and increased HRPF could be an effective treatment for respiratory diseases in adolescents. Therefore, we suggest that health practitioners should pay more attention to helping adolescents with RD gain or maintain high HRPF, and recommended HRPF guideline for adolescents with RD should be specifically developed.

## Figures and Tables

**Table 1:** Characteristics and anthropometrics of the participants.

Variable		General (*n* = 139)		RD (*n* = 139)	
		No. (%)	Mean ± SD	No. (%)	Mean ± SD
Gender	Boys	84 (60.43)		84 (60.43)	
	Girls	55 (39.57)		55 (39.57)	
Age (years)	12	38 (27.34)	13.38 ± 1.06	38 (27.34)	13.38 ± 1.06
	13	33 (23.74)		33 (23.74)	
	14	45 (32.37)		45 (32.37)	
	15	23 (16.55)		23 (16.55)	
Height (cm)	Boys		163.87 ± 6.88		163.68 ± 9.65
	Girls		160.65 ± 3.81		157.93 ± 5.72
Weight (kg)	Boys		55.62 ± 9.21		57.36 ± 13.00
	Girls		54.86 ± 6.67		53.96 ± 7.98
BMI (kg·m^−2^)	Boys		20.61 ± 2.78		21.20 ± 3.63
	Girls		21.20 ± 2.41		21.55 ± 2.64

RD: Respiratory disease, SD: Standard deviation, BMI: Body mass index. There were no significant differences in height, weight, or BMI between the general and RD groups (*p* > 0.05).

**Table 2:** Participants’ physical activity, sedentary behaviors, and health-related physical fitness.

Variable		General (*n* = 139)		RD (*n* = 139)		Cohen’s *d*
		No. (%)	Mean ± SD	No. (%)	Mean ± SD	
**Physical** **Activity**	Moderate-intensity PA (min/week)		1003.42 ± 1828.73		645.32 ± 1072.71 *	0.23
	Vigorous-intensity PA (min/week)		2078.39 ± 3329.86		1428.32 ± 2484.39	0.22
	Moderate-to-vigorous-intensity PA (min/week)		3081.81 ± 4793.37		2073.64 ± 3123.47 *	0.24
Sedentary Behaviors	Under 6 h	25 (17.99)		25 (17.99)		
	6–12 h	64 (46.04)		57 (41.01)		
	More than 12 h	46 (33.09)		54 (38.85)		
HRPF	PACER (laps)		51.78 ± 20.76		39.01 ± 16.83 ***	0.67
	Sit-ups (reps/min)		40.90 ± 11.12		36.93 ± 11.13 **	0.35
	Grip strength (left, kg)		26.24 ± 4.59		24.08 ± 7.31 **	0.35
	Grip strength (right, kg)		27.15 ± 5.17		25.06 ± 7.17 **	0.33
	Sit and reach (left, cm)		62.22 ± 11.87		58.15 ± 10.85 **	0.35
	Sit and reach (right, cm)		61.54 ± 11.83		58.09 ± 11.18 *	0.29
	Body composition (BIA, %)		23.89 ± 6.43		25.95 ± 7.86 *	0.28

RD: Respiratory diseases; SD: Standard deviation; HRPF: Health-related physical fitness; BIA: Bioelectrical impedance analysis; PACER: Progressive aerobic cardiovascular endurance run. * *p* < 0.05, ** *p* < 0.01, and *** *p* < 0.001.

## Data Availability

The datasets used and/or analyzed during the current study are available from the corresponding author on reasonable request.
